# Three-Class Mammogram Classification Based on Descriptive CNN Features

**DOI:** 10.1155/2017/3640901

**Published:** 2017-01-15

**Authors:** M. Mohsin Jadoon, Qianni Zhang, Ihsan Ul Haq, Sharjeel Butt, Adeel Jadoon

**Affiliations:** ^1^Queen Mary University of London, London, UK; ^2^Faculty of Engineering and Technology, International Islamic University Islamabad, Islamabad, Pakistan

## Abstract

In this paper, a novel classification technique for large data set of mammograms using a deep learning method is proposed. The proposed model targets a three-class classification study (normal, malignant, and benign cases). In our model we have presented two methods, namely, convolutional neural network-discrete wavelet (CNN-DW) and convolutional neural network-curvelet transform (CNN-CT). An augmented data set is generated by using mammogram patches. To enhance the contrast of mammogram images, the data set is filtered by contrast limited adaptive histogram equalization (CLAHE). In the CNN-DW method, enhanced mammogram images are decomposed as its four subbands by means of two-dimensional discrete wavelet transform (2D-DWT), while in the second method discrete curvelet transform (DCT) is used. In both methods, dense scale invariant feature (DSIFT) for all subbands is extracted. Input data matrix containing these subband features of all the mammogram patches is created that is processed as input to convolutional neural network (CNN). Softmax layer and support vector machine (SVM) layer are used to train CNN for classification. Proposed methods have been compared with existing methods in terms of accuracy rate, error rate, and various validation assessment measures. CNN-DW and CNN-CT have achieved accuracy rate of 81.83% and 83.74%, respectively. Simulation results clearly validate the significance and impact of our proposed model as compared to other well-known existing techniques.

## 1. Introduction

Recent studies show that in UK the second most leading cause of deaths due to cancer in women is breast cancer. In UK every year around 55,000 women are diagnosed with the breast cancer that is equivalent of one person every 10 minutes. One woman out of eight in her life time has a chance to be diagnosed as a sufferer of breast cancer [1]. Similar statistics are also shown in USA, with 231,000 estimated new cases for breast cancer in 2015 [2]. Breast cancer usually takes time to develop and symptoms are shown very late. As there is no effective way to cure later stage breast cancer, many lives can be saved if it can be detect at early stage. Therefore, for the early detection of breast cancer, it is recommended by America Cancer Society (ACS) that every woman who has a high risk factor of breast cancer should take screening test once in a year [2].

In current technical era, computerized diagnostic systems widely use mammogram screening methods to classify the breast tumor. Computer aided diagnosis (CAD) system typically relies on machine learning techniques to detect tumors in digitized mammogram images. Such techniques need to work with discriminant and descriptive features to classify images into multiple classes. In the past decade numerous methods have been proposed to classify the mammograms images and to attain better accuracy, efficiency, robustness, and precision. Nevertheless it is still an open research area due to the intrinsic challenges in mammogram representation and classification.

Many researchers have studied mammogram images for two-class (normal versus abnormal) classification and achieved significant results. Mazurowski et al. proposed a template based on a recognition algorithm for breast masses [3]. Their data set was based on 1,852 Digital Database for Screening Mammography (DDSM) images and achieved accuracy up to 83%. Lesniak et al. compared the performance of support vector machine (SVM) based classification with nearest neighbor algorithms [4]. They have used a private data set of mammography patches containing 10,397 images. The accuracy of their model was up to 67%. Wei et al. presented a relevance feedback learning method and performed classification using SVM radial kernel with a data set of 2,563 DDSM images [5]. Tao et al. compared the performance of two classifiers named curvature scale space and local linear embedded matric using a database of 476 and 415, and the accuracy of the two classifiers was 75% and 80%, respectively [6]. Abirami et al. [7] used wavelet features for the two-class classification of digital mammograms; they have achieved 93% accuracy on MIAS data set. Elter and Halmeyer [8] performed classification using Artificial Neural Network (ANN) and Euclidean metric classifier, respectively, and achieved a performance over 85%. All of the above researchers used two-class classification but two-class classification is not enough to avoid unnecessary biopsy because in abnormal cases the tumor can be either benign or malignant. Suckling proposed Extreme Learning Machine (ELM) method to classify mammograms of the Mammographic Images Analysis Society (MIAS) database [9]. The algorithm outperformed other techniques with same database [10]. Jasmine et al. performed two-class classification with his proposed method based on wavelet analysis using Artificial Neural Network (ANN) [11]. This experiment was performed using MIAS database of 322 images and has achieved accuracies up to 87%. In [12] Xu et al. compared the performance of three NNs and suggest that Multilayer Perceptron (MLP) performance improved as the number of features increased. They have achieved an accuracy up to 98% by using 120 mammogram images. Deserno et al. have used Image Retrieval in Medical Applications (IRMA) data set containing 2796 images, experimented based on 2D principal component analysis (2DPCA) and achieved accuracy up to 80% [13]. However, they have used 20 classes in their classification.

In the last few years, deep learning using NN has achieved state-of-the-art results in many fields of computer vision, such as object detection and classification [14]. Deep learning models are also applied on various medical imaging fields like tissue classification in histopathology and histology images [15]. However, in literature only a limited number of studies are available using deep learning for mammogram images classification [16]. In [17], CNNs were used to segment the breast tissue of mammographic texture. Multiscale features and autoencoders were applied to calculate breast density score [18]. CNNs were used to classify the microcalcifications but the data set was very small [19]. Kallenberg et al. proposed unsupervised deep learning applied to breast density segmentation [20]. Jamieson et al. used Adaptive Deconvolutional Networks (ADN) to characterize breast into malign/benign [21]. Their scheme was tested on 739 full field digital mammography (FFDM) images and 2393 ultrasound images. Arevalo et al. proposed a CNN model and achieved an accuracy up to 86% [22]. They used 736 images of BCDR-F03 data set. In [23], Mert et al. proposed radial basis function neural network (RBFNN) with independent component analysis (ICA) for two-class classification. They achieved an accuracy of 90% on the WBDC data set [24] with 569 images. Recently for two-class classification Dheeba and Abdel-Zaher et al. used Particle Swarm Optimization-based Wavelet Neural Network (PSO-WNN) and deep belief network (DBN) [25], [26], respectively, and achieved significant results on data set of 216 and 690 images. Uppal and Naseem used fusion of discrete cosine transform and discrete wavelet transform features to classify mammograms in 3 classes [27]; they used data in the MIAS database and obtained high accuracy of 96.97% and 98.39%, respectively. Deep learning methods can perform well at the cost of large amount of data set [28–30].

Table 1 summarizes the significant work done so far for the classification of mammogram images. It can be seen that significant results are achieved for two-class classification. However, for three-class (normal, benign, and malignant) classification, there has been little progress because either of the available data sets are small and private or proposed systems have not achieved very promising results.

**Figure 1:**
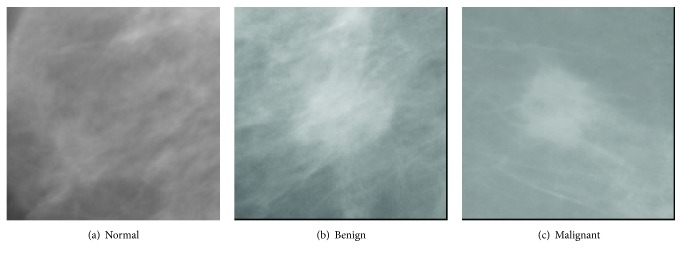
Sample images of mammogram patches.

**Figure 2:**
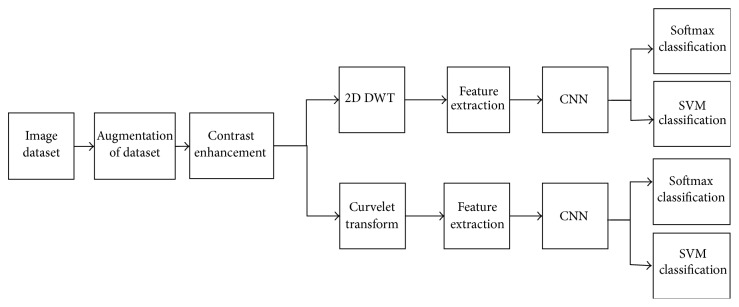
Flow chart of proposed model.

In this paper, we have extended our previous work [31] and propose an improved classification technique for large data sets of mammograms using CNN. The application of classic approaches, for example, using DSIFT features and SVM classifier, on a classic two-class classification for normal and abnormal or a three-class classification (normal, benign, and malignant) using the rotation and scale invariant DSIFT features [32] and a SVM classifier with linear kernel, did not achieve satisfactory performance. Therefore, a three-class classification study (malignant, benign, and normal) is carried out by using our proposed model. Example images of these classes are shown in Figure 1. Two different approaches, namely, CNN-DW and CNN-CT, are presented in our proposed model. An augmented data set is produced by using mammogram patches. The data set is filtered by contrast enhancement. In the first method enhanced mammogram images are decomposed as its four subbands by means of 2D-DWT, while in the second method discrete curvelet transform (DCT) is used. In both methods DSIFT descriptor is used to extract features for all subbands. Input data matrix containing these subband features of all the mammogram patches is created that is processed as input to convolutional neural network (CNN). A softmax layer and a SVM layer are used to train CNN for classification. A flow chart of the proposed model is given in Figure 2.

The main contribution of this paper is the development of a deep learning method based on a large data set of mammogram images. We have shown that the discriminant and descriptive features can perform well with different wavelets, if these are used according to our proposed model in combination with CNN. We also perform classification with SVM via 10-fold cross-validation presenting more unbiased results.

The remaining of the paper is organized as follows.  Section 2 explains the feature extraction and representation steps in this research.  Section 3 describes the CNN based classification model and SVM classification.  Section 4 demonstrates the simulation/results and the paper concludes in Section 5.

## 2. Feature Extraction and Representation

### 2.1. Data Augmentation

In deep learning techniques, the NN models need to learn a large number of parameters. The chance of overfitting the training data increases due to the model complexity. Augmentation of data is an upright way to avoid this action [33]. It artificially creates new sample images by applying transformations like flipping, rotation, and many other makeovers to the actual data sample. For every image, artificially we have produced seven new sample images using the combination of 90, 180, and 270 degrees of rotation and flipping transformations. Thus, the resulting data set contains seven times more images than the original database has.

### 2.2. Enhancement of Digital Mammograms

Contrast Limited Adaptive Histogram Equalization (CLAHE) method [36] is used to enhance the often degraded contrast in some of mammogram images. The pixel intensity transforms to a value within the display range proportional to the pixel intensity's rank in the local intensity histogram. CLAHE is a special case of Adaptive Histogram Equalization (AHE) where images are enhanced by a user defined clip level, that is, height of the local histogram, and thus on the maximum contrast enhancement factor. In this technique, enhancement is done on very small patches, so the overenhancement due to noise or the effect of edge-shadowing is very low as compared to AHE [37].

The CLAHE method was originally developed to reduce the shadow of edges and noise produced in homogeneous areas in medical images [38]. The method has been used for the enhancement of digital mammograms [36–40] and demonstrated good improvements to mammograms visual quality.

An input image *I* with dimensions *M* × *N*, is divided into small blocks. CLAHE is then used to enhance the contrast of each block. Finally the bilinear interpolation is used to combine the neighboring blocks back into whole images. The steps in CLAHE are described as below [40].

(1) Images patches are divided into nonoverlapping blocks of size 8 × 8.
(2) The histogram of each block is calculated.
(3) For contrast enhancement of patches, a clip limit of histogram, *t* = 0.001, is set.
(4) After clipping the threshold value the histogram is redistributed.
(5) Every block histogram is modified by the following transformation function:
  (1) $$\begin{array}{c} A_{t}=\overset{t}{\underset{i=0}{\sum }}p_{t}\left(\;A_{i}\;\right), \end{array}$$
where *p*_*t*_(*A*_*i*_) is the probability density function of the input patch image grayscale value at *i* and is define as
  (2) $$\begin{array}{c} p_{t}\left(\;A_{i}\;\right)=\frac{m_{i}}{m}, \end{array}$$
where *m*_*i*_ is the gray scale value of input pixel *i* and *m* is the total number of pixels in a block.
(6) Bilinear interpolation is used to combine the neighboring blocks in each patch. The gray scale value of the patch is also changed according to the new histogram.

In our experiment, we have used the block size of 8 × 8 and clip limit of histogram is defined as 0.001.

### 2.3. Two-Dimensional Discrete Wavelet Transform

**Figure 3:**
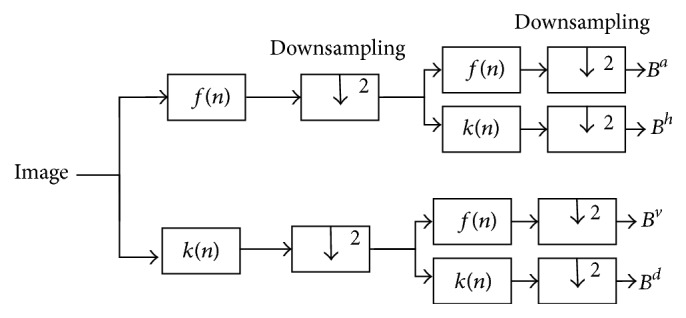
Two-dimensional discrete wavelet transform.

A two-dimensional DWT consists of downsamplers and digital filter banks. The digital filter banks comprise low pass filter *f*(*n*) and high pass filter *k*(*n*). The number of banks depends upon desired resolution of the application [41]. As the mammogram images are two-dimensional signal, the DWT can be computed by separable wavelet functions. As shown in Figure 3, the columns and rows of the image are distinctly processed over the one-dimensional wavelet transform to establish the two-dimensional DWT. In frequency domain the enhanced image *E* is decomposed into subband images at resolution 2^*j*+1^. *B*^*a*^ is the approximation of the image. *B*^*d*^, *B*^*h*^, and *B*^*v*^ are three detailed subband images in diagonal, horizontal, and vertical, directions, respectively.

As a result of wavelet decomposition the image *I* decomposed into four subband components like High-High (HH), High-Low (HL), Low-High (LH), and Low-Low (LL), which correspond to subimages that are *B*^*a*^, *B*^*d*^, *B*^*v*^, and *B*^*h*^, respectively, as shown in Figure 3.

### 2.4. Discrete Curvelet Transform

Discrete curvelet transform is an image representation technique used in computer vision. It was proposed by Candes and Donoho [42]. DCT codes image edges more efficiently than wavelet transform [43] and it has useful geometric features that can be used as a feature vector in medical image processing. Eltoukhy et al. [44, 45] have used DCT for the mammogram images.

Let *L* be a function that has a discontinuity across a curve and is smooth otherwise, and consider approximating *L* from the best *n*-terms in the expansion. The squared error of such an *n*-term expansion obeys [46]

(3) $$\begin{array}{c} {\left\|\;L-L_{\tilde{f}}\;\right\|}^{2}\alpha \frac{1}{\sqrt{m}},\;m⟶+\infty , \end{array}$$

where $L_{\tilde{f}}$ is the approximation from *n* best Fourier coefficients. Equation (4) shows the expansion for wavelet,

(4) $$\begin{array}{c} {\left\|\;L-L_{\tilde{w}}\;\right\|}^{2}\alpha \frac{1}{m},\;m⟶+\infty , \end{array}$$

where $L_{\tilde{w}}$ is the approximation from *n* best wavelet coefficients.

Equation (5) shows the expansion for curvelet expansion,

(5) $$\begin{array}{c} {\left\|\;L-L_{\tilde{c}}\;\right\|}^{2}\alpha \frac{1}{m^{2}}\log{\left(\;m\;\right)}^{3},\;m⟶+\infty , \end{array}$$

where $L_{\tilde{c}}$ is the approximation from the *n* best curvelet coefficients.

Equation (5) shows that the MSE will be reduced in DCT. Fast DCT proposed in [47] is described as below.

It has a two-dimensional space *R*^2^ with *ω* as the frequency domain variable and *x* as the spatial variable, and *r* and *θ* are the polar coordinates in the frequency domain. A pair of windows *V*(*t*) and *W*(*r*) are defined, which will be called the angular window and the radial window, respectively. *V* is taking real arguments and supported on *r* ∈ (−1,1) and *W* is taking positive real arguments and supported on *r* ∈ (1/2,2).

(6) ∑a=−∞∞w22ar=1,r∈32,32,∑b=−∞∞v2t−b=1,r∈−12,12.

For each *a* ≥ *a*_0_, a frequency window *U*_*a*_ is defined as

(7) $$\begin{array}{c} U_{a}\left(\;r,\theta \;\right)=2^{-3a/4}w\left(\;2^{-a}r\;\right)v\left(\;\frac{2^{\left(\varepsilon j/2\right)\theta }}{2\pi }\;\right). \end{array}$$

The scaled and shifted curvelet in frequency domain is defined as

(8) $$\begin{array}{c} {\tilde{\varphi }}_{a,k,b}\left(\;x\;\right)={\tilde{\varphi }}_{j}\left(\;U_{a}\left(\;r-k,\theta -\theta_{b}\;\right)\;\right). \end{array}$$

From Plancherel theorem, curvelet coefficients can be computed as

(9) $$\begin{array}{c} C_{a,k,b}\left(\;x\;\right)=\frac{1}{{\left(2\pi \right)}^{2}}\int f\left(\;\omega \;\right){\tilde{\varphi }}_{a}\left(\;U_{a}\left(\;r-k,\theta -\theta_{b}\;\right)\;\right)d\omega . \end{array}$$

*C*_*a*,*k*,*b*_(*x*) are curvelet coefficients in 4 subbands of spatial frequencies, namely, *F*1, *F*2, *F*3, and *F*4.

### 2.5. Dense Scale Invariant Feature Transform

In next step DSIFT descriptor is extracted from all the subbands components. Dense SIFT scale-space extrema detection used Difference-of-Gaussian (DOG) function to identify potential interest points [48], which were invariant to scale and orientation.

(10) $$\begin{array}{c} D\left(\;x,y,\sigma \;\right)=\left(\;G\left(\;x,y,\alpha \sigma \;\right)-G\left(\;x,y,\sigma \;\right)\;\right)*\tilde{E}\left(\;x,y\;\right), \end{array}$$

where *α* is a constant multiplicative factor, $\tilde{E}$ is the decomposed subband of enhanced patch *E*, and *G*(*x*, *y*, *σ*) represent variable scale Gaussian; that is,

(11) $$\begin{array}{c} G\left(\;x,y,\sigma \;\right)=\frac{1}{2\pi \sigma^{2}}e^{-\left(x^{2}+y^{2}/2\sigma^{2}\right)}. \end{array}$$

Equation (10) can be written as

(12) Dx,y,σGx,y,ασ∗Ix,y−Gx,y,σ∗E~x,y=Lx,y,ασ−Lx,y,σ,

where the scale space of an image *L*(*x*, *y*, *ασ*) is the convolution of *G* with an input image $\tilde{E}(x,y)$. DOG is used here instead of Gaussian to improve the computation speed.

In the key point localization stage, Hessian matrix is used to compute principal curvatures that eliminate the edges by rejecting the low contrast point [48]. Key point descriptor can be found out by using a three-dimensional histogram in which two dimensions correspond to image spatial dimensions and the third dimension corresponds to the image gradient direction computed centered at the key points.

The DSIFT descriptor is applied to all the subbands with step size 4 and radius size 5. Feature matrices having dimension (128 × 400) are extracted for all the subbands. From the columns of this matrix, six time domain features, kurtosis, mean, skewness, energy, maximum, and standard deviation, are extracted for each subband. The resultant feature matrix is of the shape of (128 × 6). This matrix is reshaped into a vector form of (1 × 768). Weighting coefficients are applied to the subband images according to (13) and (14) for CNN-DW and CNN-CT method, respectively.

(13) Feature  vector=3∗LL+2∗LH+2∗HL,

(14) Feature  vector=F1+3∗F2+2∗F3+2∗F4.

Equal zero padding is performed on the start and end columns such that it reshapes as (1 × 785). Enhancement and feature extraction steps are performed on all the augmented data sets so that we have a data matrix $\tilde{M}(x,y)$ of the shape (22368 × 785), where 22368 is the number of the sample images and 784 is the number of features of each sample, and every sample has a last column label that belongs to its receptive patch class.

## 3. Convolution Neural Network

In the next step we use CNN to learn features from the data set matrix $\tilde{M}$. CNN has proved its importance in classification of images by its significance results. CNN has a multilayered architecture, consisting of a convolution layer followed by a maximum pooling layer. The number of layers depends upon the designer. The output of final maximum pooling layer is fed to a fully connected layer that works like MLP which is further forwarded to softmax layer.

The convolution layer takes 1D or 2D matrices as an input. Equation (15) shows the single output matrix of convolution layer.

(15) $$\begin{array}{c} C_{j}=f\left(\;\overset{N}{\underset{i=1}{\sum }}{\tilde{M}}_{i}*L_{i,j}+\rho_{j}\;\right), \end{array}$$

where ${\tilde{M}}_{i}$ is the input matrix that convolves with kernel matrices *L*_*i*,*j*_. Bias *ρ*_*j*_ is added to each element of output after computing the sum of all convoluted matrices. *C*_*j*_ is the one output matrix computed by a nonlinear activation function *f*, that is applied to each element. Commonly used activation functions in convolution layer are tangent hyperbolic function and sigmoid function as follows:

(16) fx=11+exp⁡−x,fx=tanhx.

The pooling layer is used for dimensionality reduction in the convolution layer. Mostly used pooling layer algorithms are average pooling, mean pooling, and maximum pooling. During the training, the dropout algorithm is applied by randomly disabling the neurons, with a normally dropout ratio between 0.3 and 0.6. The final layer of CNN is a soft max layer that contains the output neuron according to the number of classes of the problem, which is assigned a confidence score.

**Figure 4:**
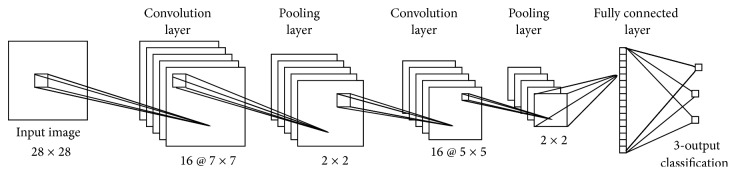
Convolution neural network model.

The overall network design of CNN is presented in Figure 4. The two convolutional and max pooling layers are used with a kernel size of 2 × 2. Convolutional layers have 16 kernels with size of 7 × 7 and the second layer uses kernel sized 5 × 5. Then, a fully connected neural layer is used. The dropout ratio in the experiment is 0.55. Softmax layer is used to train CNN for classification.

### 3.1. Classification with Support Vector Machines

Recently, many researchers have used SVM as a top layer instead of softmax layer in deep learning and showed improvements in the classification result [49]. In the second experiment we also use SVM layer instead of the softmax layer. All the other settings of the process remain the same as explained above.

SVMs have been applied to many classification tasks [50, 51]. Input data *x* is labeled as *y* = −1 for class 1 and as *y* = 1 for class 2. For linearly separable data a hyperplane can be defined as

(17) $$\begin{array}{c} g\left(\;x\;\right)=w^{T}x+b=\overset{n}{\underset{i=1}{\sum }}w_{i}^{T}x_{i}+b, \end{array}$$

where *x* is the input vector, *b* is a scalar, and *w* is *n* dimensional normal vector of this hyperplane. Distance from origin perpendicular to this plane is −*b*/‖*w*‖. The solution of SVM is based on optimal hyperplane and minimum mean square error that is defined as

(18) $$\begin{array}{c} E\left(\;w,b\;\right)=\frac{1}{2}{\left\|\;w\;\right\|}^{2}-\overset{m}{\underset{i=1}{\sum }}\lambda_{i}{\left(\;y_{i}-g\left(\;x_{i}\;\right)\;\right)}^{2}, \end{array}$$

where *λ* is a Lagrangian coefficient and *λ*_*i*_ > 0. Maximizing (18) results,

(19) w=∑i=1mλiyixi,∑i=1mλiyi=0.

Putting (19) into (18), it is redefined as

(20) $$\begin{array}{c} \nu \left(\;\lambda \;\right)=\overset{m}{\underset{i=1}{\sum }}\lambda_{i}-\frac{1}{2}\overset{m}{\underset{i=1}{\sum }}\;\overset{m}{\underset{j=1}{\sum }}\lambda_{i}\lambda_{j}y_{i}y_{j}K\left(\;x_{i}x_{j}\;\right), \end{array}$$

where *K*(*x*_*i*_*x*_*j*_) is the kernel function [52].

## 4. Simulation and Results

This section presents the database and validation assessment measures that are used in this experiment. Moreover, the experimental results are presented to show the superiority of proposed methods.

### 4.1. Database

We have used IRMA data set [53] for experiments in this study. A total of 2796 patches of the original mammogram images are used for this experiment. Selected IRMA patches consist of four different sources including 2,576 images from DDSM, 150 images from MIAS, and Lawrence Livermore National Laboratory (LLNL) and Rheinisch Westfälische Technische Hochschule (RWTH) contribute 1 and 69 images, respectively. The selected images are further divided into three classes, malignant, benign, and normal, as prescribed in IRMA data set. The final size of mammogram images patches is 128 × 128 pixels.

### 4.2. Validity Assessment Measures

The validation of the method is measured by classification accuracy, Positive Predictive Value (PPV), Negative Predictive Value (NPV), sensitivity, specificity, Matthews Correlation Coefficient (MCC), and Receiver Operating Characteristic (ROC).

In medical image classification, false positive (FP) is the incorrect classification rate of samples, such that a disease result is positive, when in reality it is not, while false negative (FN) is the incorrect classification rate of samples, in which a test result improperly indicates no presence of a condition. True positive (TP) is the correct classification rate of positive samples, while true negative is the correct classification rate of negative samples.

Accuracy is the most commonly used assessment measure for classification that considers all the cases; it used all the cases.

(21) $$\begin{array}{c} Accuracy=\frac{TN+TP}{FP+FN+TP+TN}. \end{array}$$

PPV is defined as the number of the correct detected positive cases over all detected positive cases.

(22) $$\begin{array}{c} PPV=\frac{TP}{FP+TP}. \end{array}$$

NPV is defined as the number of the true negative cases detected over all negative cases.

(23) $$\begin{array}{c} NPV=\frac{TN}{FN+TN}. \end{array}$$

Sensitivity is defined as the ratio of the detected true positive cases over actual positive cases. It deals only with positive cases.

(24) $$\begin{array}{c} Sensitivity=\frac{TP}{FN+TP}. \end{array}$$

Unlike sensitivity, specificity deals only with negative cases. It is the ratio of the detected true negative over the actual negative.

(25) $$\begin{array}{c} Specificity=\frac{TN}{FP+TN}. \end{array}$$

MCC is an assessment indicator of deep learning methods, particularly for the negative case sample detected, that are evidently unbalanced compared with the positive sample detected. MCC provides a superior assessment compared to the general accuracy.

(26) MCC=TP×TN−FP×FNTP+FPTP+FNTN+FPTN+FN.

The ROC curve is used for measuring the predictive accuracy of the model. It indicates the relation between the true positive rate and false positive rate.

### 4.3. Experimental Results

**Figure 5:**
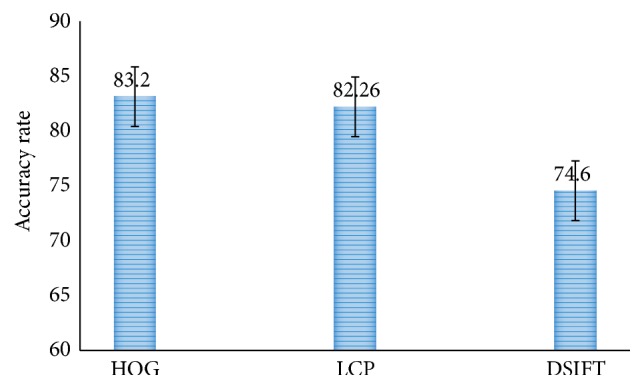
Comparison of two-class classification accuracy rate for HOG LCP and DSIFT.

**Figure 6:**
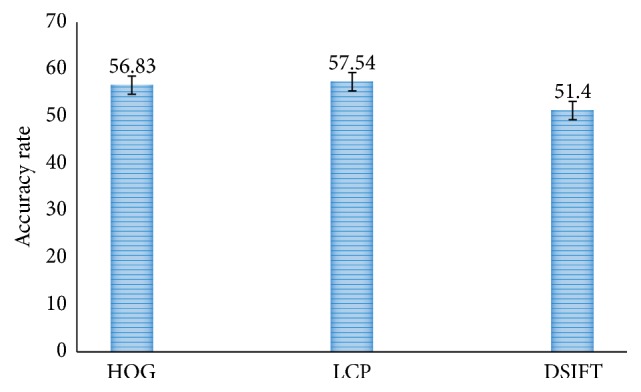
Comparison of three-class classification accuracy rate for HOG LCP and DSIFT.

In this subsection the proposed methods have been compared with existing methods in terms of accuracy rate, error rate, and various validation assessment measures.  Figure 5 shows the result of two-class classification. It can be observed that in two-class classification Histogram Oriented Gradient (HOG) method performs better with an accuracy rate of 83.2%. The other two schemes, Local Configuration Pattern (LCP) and DSIFT, have accuracy rates of 82.26% and 74.6%, respectively. Likewise, Figure 6 shows the result of three-class classification. Here LCP method performs better than the other two schemes with the best accuracy of 57.54, but the results are not so promising. This accuracy has been further enhanced by our methods as shown in the rest of the simulation results.

**Figure 7:**
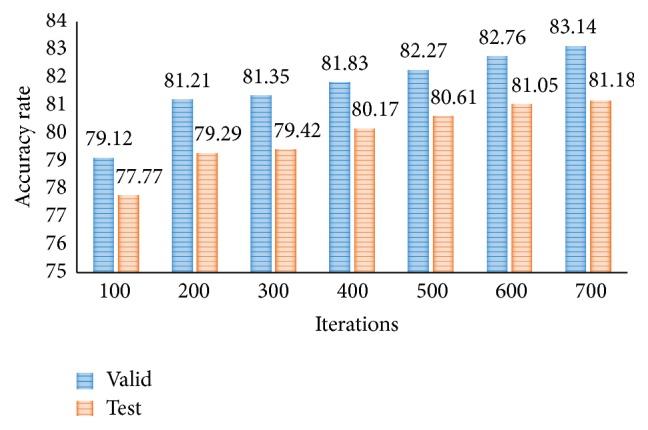
Accuracy rate of proposed CNN-DW method for test and validation data sets.

**Figure 8:**
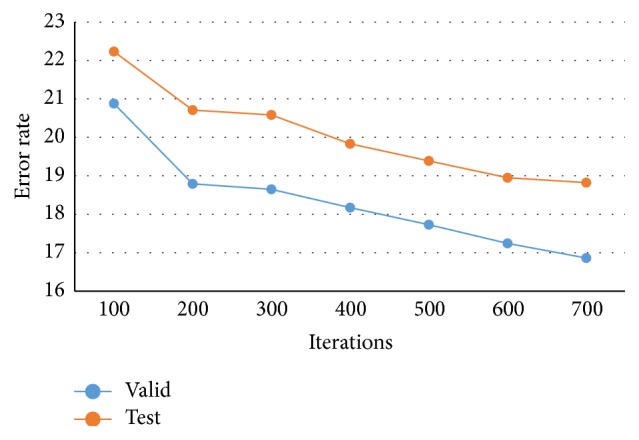
Error rate of proposed CNN-DW method for test and validation data sets.

In Figure 7, the accuracy rate of proposed CNN-DW method has been presented for different number of iterations using softmax layer. Note that the classification results for three-class category obtained by proposed CNN-DW method are more pleasing as compared to the existing schemes in Figure 6. CNN-DW method achieved the accuracy of 83.14% and 81.18% on validation data set and test data set, respectively. Furthermore, Figure 8 shows the error rate of the proposed CNN-DW method with softmax layer at different iterations. With softmax layer, it has 16.86 and 18.82 error on validation data set and test data set, respectively.

**Figure 9:**
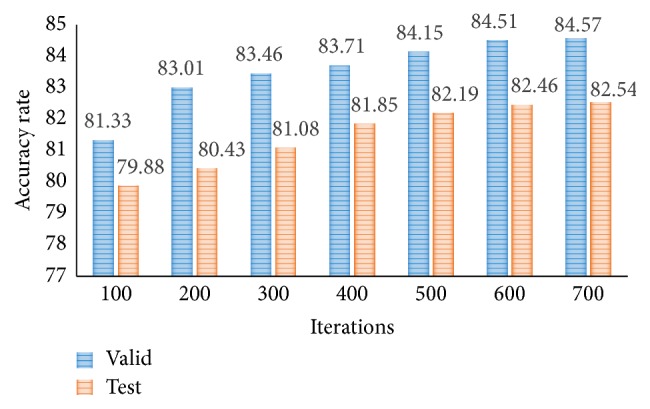
Accuracy rate of proposed CNN-CT method for test and validation data sets.

**Figure 10:**
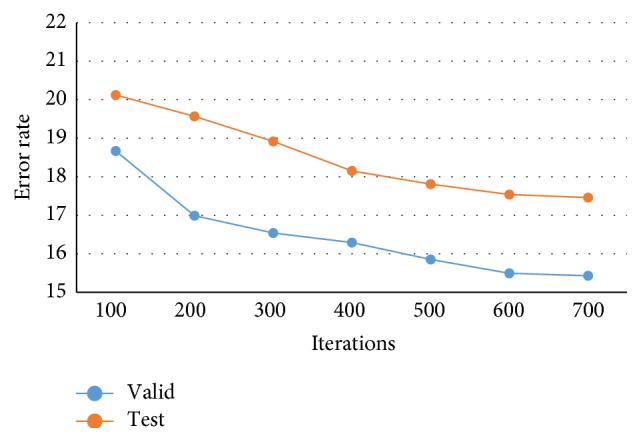
Error rate of proposed CNN-CT method for test and validation data sets.

Likewise, the accuracy rate and error rate of second proposed method, that is, CNN-CT, have been shown.  Figure 9 shows the accuracy rate of proposed CNN-CT method with softmax layer at different iterations. Note that the classification results for three-class category obtained by proposed CNN-CT method are better as compared to the existing schemes in Figure 6 and from CNN-DW method as well. The proposed method achieved the accuracy of 84.57% and 82.54% on validation data set and test data set, respectively. Similarly, Figure 10 shows the error rate of proposed CNN-CT method with softmax layer at different iterations. With softmax layer, it has 15.43 and 17.46 error on validation data set and test data set, respectively.

**Figure 11:**
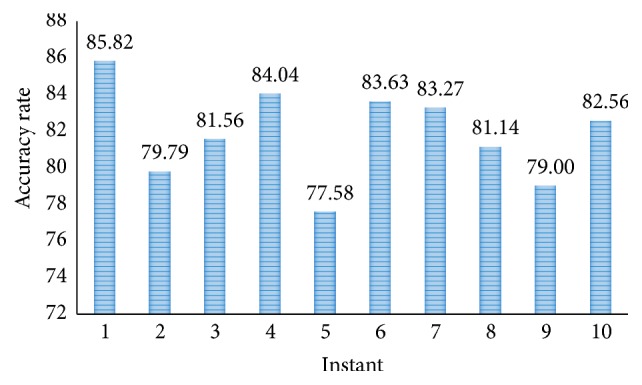
Accuracy rate of proposed CNN-DW method with SVM classifier.

In the further simulation, the results of our proposed methods using SVM layer are presented.  Figure 11 shows the accuracy rate of proposed CNN-DW method with SVM layer at different instants. It is shown that proposed CNN-DW method has achieved an average accuracy of 81.83%. Likewise, Figure 13 shows the accuracy rate of the other proposed CNN-CT method with SVM layer. Proposed curvelet method has achieved average accuracy of 83.74%.

**Figure 12:**
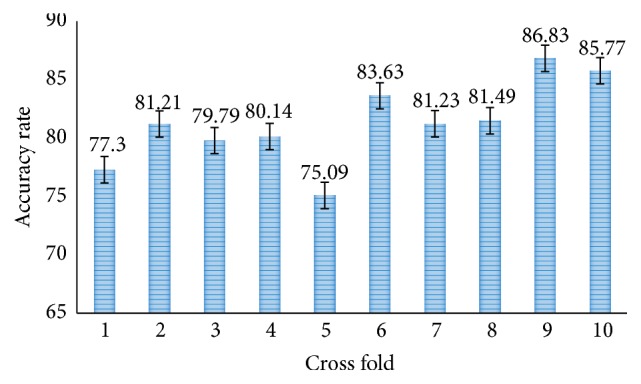
Accuracy rate of proposed CNN-DW method with SVM using 10-fold cross-validation.

**Figure 13:**
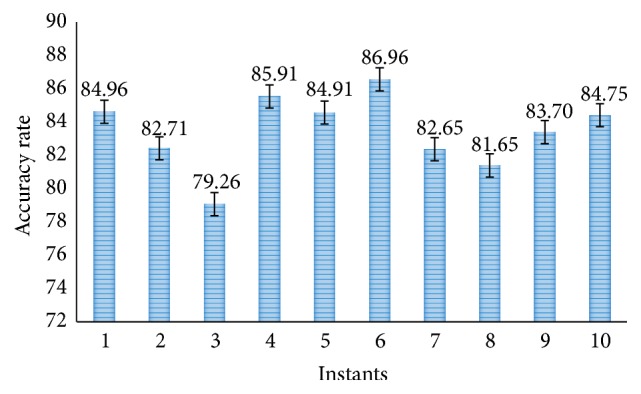
Accuracy rate of proposed CNN-CT method with SVM classifier.

**Figure 14:**
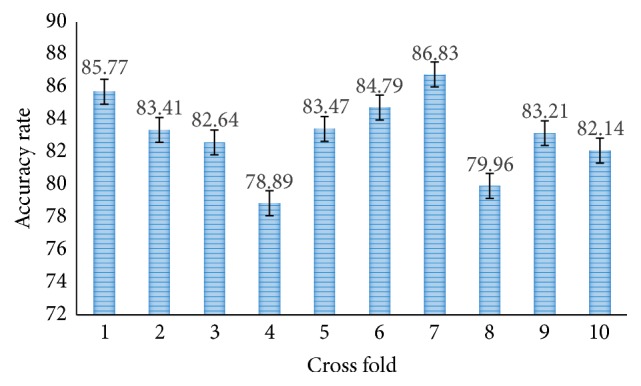
Accuracy rate of proposed CNN-CT method with SVM using 10-fold cross-validation.

Moreover, the proposed methods are also tested for SVM 10-fold cross-validation.  Figure 12 shows the accuracy rate of proposed CNN-DW method with SVM layer and it has achieved average accuracy of 81.23% in 10-fold cross-validation. Similarly, Figure 14 shows the accuracy rate of proposed CNN-CT method with 10-fold cross-validated SVM layer. It has achieved an average accuracy of 83.11%.

Table 2 shows the quantitative comparison of existing and proposed schemes. It can easily be observed that the proposed CNN-DW and CNN-CT methods provide better measure values, especially on large data set of mammogram images. Proposed CNN_WT method has outperforms all other methods. Similarly, Table 3 shows the quantitative comparison for SVM classifier with 10-fold cross-validation of the existing and proposed schemes. It can easily be observed that the proposed scheme provides better measure values in both the cases. Finally, Table 4 provides a summary on accuracy rate for 3-class classification.

## 5. Conclusion

A novel mammograms classification method for breast cancer detection based on CNN is proposed. We have proposed two algorithms; first algorithm is based on 2D discrete wavelet transform while the other is based on curvelet transform. We have found that deep learning method can be used for the breast cancer detection by using data augmentation and results show that learning features from the data set before inputting the data to the CNN is more helpful for cancer detection. We have also found that by using the SVM layer instead of softmax layer the classification performance can be improved. However, the 10-fold cross-validated result of the SVM can cut down the accuracy because the cross-validated result is more unbiased than performing training and testing process proposed method with curvelet transform has better results as compared to the proposed method with wavelet method and other existing methods. In future work, more techniques of deep learning can be applied for the detection of breast cancer. Improvement can also be made by using different architecture of CNN.

## Figures and Tables

**Table 1:** State-of-the-art diagnostic schemes for the screening mammography classification.

Authors	Year	Data sources	Technique/classifier	Classes	Number of images	Classification accuracy
Mazurowski et al. [3]	2011	DDMS	Random mutation hill climbing	2	1,852	49%–83%
Lesniak et al. [4]	2011	Private	SVM radial Kernel	2	10,397	66%-67%
Wei et al. [5]	2011	DDSM	SVM radial Kernel	2	2,563	72%–74%
Abirami et al. [7]	2016	MIAS	Wavelet features	2	322	93%
Tagliafico et al. [34]	2009	Private	Thresholding	4	160	80%–90%
Subashini et al. [35]	2010	Private	SVM radial Kernel	3	43	95%
Elter and Halmeyer [8]	2008	DDSM	Euclidean metric	2	360	86%
Deserno et al. [13]	2011	IRMA	SVM Gaussian Kernel	12	2796	80%
Tao et al. [6]	2011	Private	Local linear embedding metric	2	476	80%
			Curvature scale space		415	75%
Ge et al. [19]	2006	Private	CNN and LDA	2	196	—
		MIAS	CNN and LDA		216	—
Jamieson et al. [21]	2012	FFDM	ADN and SVM	2	739	—
		Ultrasound	ADN and SVM		2393	—
Arevalo et al. [22]	2015	BCDR-F03	CNN and SVM	2	736	79.9%–86%
Mert et al. [23]	2015	WBDC	ICA and RBFNN	2	569	90%
Dheeba et al. [25]	2015	Private	PSOWNN	2	216	93.6%
Abdel-Zaher and Eldeib [26]	2015	WBCD	DBN	2	690	99.6%
Vani et al. [10]	2010	MIAS	ELM			
Jasmine et al. [11]	2009	MIAS	Wavelet & ANN	2	322	87%
Xu et al. [12]	2008		MLPNN		120	98%
Uppal and Naseem [27]	2016	MIAS	Fusion of cosine transform	3	322	96.97%

**Table 2:** Validity assessment measures for SVM classifier.

	PPV	NPV	Sensitivity	Specificity	MCC	ROC
HOG	0.698	0.890	0.838	0.710	.671	.729
LCP	0.701	0.911	0.816	0.762	.701	.746
DSIFT	0.484	0.851	0.808	0.682	.629	.684
Proposed CNN-WT	0.853	0.921	0.876	0.819	.816	.846
Proposed CNN-CT	0.881	0.939	0.888	0.801	.829	.855

**Table 3:** Validity assessment measures for SVM classifier with 10-fold cross-validation.

	PPV	NPV	Sensitivity	Specificity	MCC	ROC
HOG	0.654	0.847	0.803	0.671	.641	.687
LCP	0.663	0.881	0.769	0.737	.675	.712
DSIFT	0.441	0.824	0.776	0.642	.589	.651
Proposed CNN-WT	0.818	0.891	0.833	0.782	.802	.831
Proposed CNN-CT	0.839	0.904	0.854	0.797	.810	.839

**Table 4:** Summarized accuracy rate for 3-class classification on large data set.

	HOG	LCP	DSIFT	CNN-WT	CNN-CT
SVM layer	56.83	57.54	51.40	81.83	83.74
SVM 10-fold cross-validation	56.27	57.13	50.91	81.24	83.11
Softmax layer	—	—	—	79.92	81.49
